# Supplementary material for: Shift in Metabolite Profiling and Mineral Composition of Edible Halophytes Cultivated Hydroponically Under Increasing Salinity
Source: Metabolites. 2025 Nov 5;15(11):724. doi: 10.3390/metabo15110724 (PMC12654821; doi:10.3390/metabo15110724)
Supplement: Supplementary file 1 [file metabolites-15-00724-s001.zip › metabolites-3942346-supplementary.pdf]

Table S1. Correlation matrix (Pearson), *r*-values and *p*-values in *Plantago coronopus*.

Variables		Salinity	Fresh Weight	Dry Weight	Dry Matter	Fructose	Glucose	Raffinose	Oxalic acid	Malic acid	Fumaric acid	Succinic acid	Ascorbic acid	Citric acid	Total Proteins	Violaxan thin	Lutein	b-carotene	P	K	Ca	Mg	Fe	Mn	Na	Zn
															***p*-values**											
Salinity			**-0.923**	**-0.897**	**0.742**	**0.731**	**0.952**	**0.882**	-0.346	**-0.977**	**-0.962**	**-0.945**	**-0.934**	-0.210	**-0.907**	**0.758**	**-0.546**	0.140	0.388	**-0.845**	**-0.804**	**-0.613**	0.132	-0.047	**-0.773**	**0.656**
Fresh Weight		**<0.0001**		**0.986**	**-0.660**	**-0.623**	**-0.969**	**-0.683**	0.237	**0.963**	**0.969**	**0.826**	**0.966**	-0.035	**0.778**	**-0.906**	0.312	-0.400	-0.326	**0.931**	**0.877**	**0.718**	-0.153	0.099	**0.864**	**-0.568**
Dry Weight		**<0.0001**	**<0.0001**		**-0.546**	**-0.590**	**-0.952**	**-0.644**	0.134	**0.947**	**0.956**	**0.809**	**0.964**	-0.034	**0.753**	**-0.890**	0.270	-0.419	-0.285	**0.925**	**0.885**	**0.730**	-0.113	0.122	**0.859**	**-0.526**
Dry Matter		**0.002**	**0.007**	**0.035**		**0.690**	**0.662**	**0.719**	**-0.582**	**-0.697**	**-0.665**	**-0.701**	**-0.582**	-0.209	**-0.706**	0.506	**-0.640**	-0.108	0.261	**-0.584**	**-0.537**	-0.413	0.112	-0.093	-0.457	0.498
Fructose		**0.002**	**0.013**	**0.021**	**0.004**		**0.674**	**0.632**	-0.087	**-0.719**	**-0.666**	**-0.671**	**-0.546**	-0.244	**-0.664**	0.390	**-0.613**	-0.178	0.159	-0.460	-0.484	-0.320	0.020	-0.127	-0.369	0.433
Glucose		**<0.0001**	**<0.0001**	**<0.0001**	**0.007**	**0.006**		**0.708**	-0.279	**-0.986**	**-0.992**	**-0.832**	**-0.972**	0.036	**-0.786**	**0.868**	-0.322	0.385	0.402	**-0.915**	**-0.860**	**-0.685**	0.222	-0.038	**-0.883**	**0.634**
Raffinose		**<0.0001**	**0.005**	**0.009**	**0.002**	**0.012**	**0.003**		-0.430	**-0.771**	**-0.731**	**-0.951**	**-0.721**	**-0.549**	**-0.901**	0.491	**-0.766**	-0.236	0.380	**-0.580**	**-0.556**	-0.373	0.036	0.027	-0.512	**0.621**
Oxalic acid		0.207	0.394	0.634	**0.023**	0.759	0.314	0.110		0.267	0.264	0.388	0.240	0.225	0.410	-0.257	0.287	-0.051	-0.200	0.333	0.303	0.321	-0.074	0.072	0.341	-0.200
Malic acid		**<0.0001**	**<0.0001**	**<0.0001**	**0.004**	**0.003**	**<0.0001**	**0.001**	0.336		**0.994**	**0.878**	**0.969**	0.041	**0.836**	**-0.829**	0.421	-0.273	-0.369	**0.903**	**0.848**	**0.659**	-0.174	0.060	**0.811**	**-0.640**
Fumaric acid		**<0.0001**	**<0.0001**	**<0.0001**	**0.007**	**0.007**	**<0.0001**	**0.002**	0.342	**<0.0001**		**0.855**	**0.978**	0.005	**0.821**	**-0.855**	0.361	-0.347	-0.340	**0.935**	**0.882**	**0.709**	-0.155	0.096	**0.848**	**-0.601**
Succinic acid		**<0.0001**	**0.000**	**0.000**	**0.004**	**0.006**	**0.000**	**<0.0001**	0.154	**<0.0001**	**<0.0001**		**0.843**	0.440	**0.936**	**-0.653**	**0.681**	0.050	-0.277	**0.754**	**0.745**	**0.576**	0.051	0.128	**0.651**	**-0.553**
Ascorbic acid		**<0.0001**	**<0.0001**	**<0.0001**	**0.023**	**0.035**	**<0.0001**	**0.002**	0.389	**<0.0001**	**<0.0001**	**<0.0001**		-0.041	**0.769**	**-0.886**	0.303	-0.380	-0.419	**0.924**	**0.847**	**0.662**	-0.221	-0.008	**0.860**	**-0.654**
Citric acid		0.452	0.902	0.903	0.454	0.380	0.898	**0.034**	0.420	0.884	0.987	0.100	0.885		**0.519**	0.131	**0.683**	**0.570**	0.130	-0.068	0.034	0.048	0.390	0.221	-0.096	0.071
Total Proteins	***r*-values**	**<0.0001**	**0.001**	**0.001**	**0.003**	**0.007**	**0.001**	**<0.0001**	0.129	**0.000**	**0.000**	**<0.0001**	**0.001**	**0.047**		**-0.607**	**0.676**	0.014	-0.144	**0.751**	**0.758**	**0.634**	0.132	0.266	**0.617**	-0.408
Violaxanthin		**0.001**	**<0.0001**	**<0.0001**	0.054	0.151	**<0.0001**	0.063	0.355	**0.000**	**<0.0001**	**0.008**	**<0.0001**	0.642	**0.016**		-0.068	**0.601**	0.376	**-0.882**	**-0.784**	**-0.670**	0.264	-0.006	**-0.869**	0.508
Lutein		**0.035**	0.258	0.330	**0.010**	**0.015**	0.241	**0.001**	0.299	0.118	0.186	**0.005**	0.273	**0.005**	**0.006**	0.811		**0.702**	-0.179	0.180	0.188	0.032	0.053	-0.002	0.028	-0.412
b-carotene		0.618	0.140	0.120	0.702	0.527	0.156	0.396	0.857	0.324	0.204	0.861	0.163	**0.026**	0.960	**0.018**	**0.004**		0.026	**-0.530**	-0.497	**-0.570**	0.125	-0.156	**-0.645**	-0.059
P		0.152	0.236	0.302	0.348	0.572	0.137	0.163	0.475	0.176	0.215	0.318	0.120	0.644	0.608	0.167	0.524	0.928		-0.125	0.048	0.268	**0.914**	**0.879**	-0.388	**0.917**
K		**<0.0001**	**<0.0001**	**<0.0001**	**0.022**	0.085	**<0.0001**	**0.023**	0.225	**<0.0001**	**<0.0001**	**0.001**	**<0.0001**	0.810	**0.001**	**<0.0001**	0.522	**0.042**	0.656		**0.967**	**0.878**	0.014	0.311	**0.868**	-0.363
Ca		**0.000**	**<0.0001**	**<0.0001**	**0.039**	0.068	**<0.0001**	**0.031**	0.273	**<0.0001**	**<0.0001**	**0.001**	**<0.0001**	0.904	**0.001**	**0.001**	0.503	0.060	0.865	**<0.0001**		**0.954**	0.206	0.488	**0.843**	-0.196
Mg		**0.015**	**0.003**	**0.002**	0.126	0.245	**0.005**	0.171	0.243	**0.008**	**0.003**	**0.025**	**0.007**	0.866	**0.011**	**0.006**	0.909	**0.026**	0.334	**<0.0001**	**<0.0001**		0.382	**0.668**	**0.761**	0.080
Fe		0.638	0.586	0.687	0.691	0.943	0.426	0.898	0.792	0.534	0.581	0.856	0.428	0.151	0.640	0.342	0.852	0.658	**<0.0001**	0.961	0.462	0.160		**0.895**	-0.235	**0.770**
Mn		0.868	0.725	0.664	0.742	0.652	0.894	0.924	0.798	0.831	0.733	0.650	0.978	0.428	0.338	0.982	0.995	0.580	**<0.0001**	0.260	0.065	**0.006**	**<0.0001**		0.048	**0.708**
Na		**0.001**	**<0.0001**	**<0.0001**	0.086	0.176	**<0.0001**	0.051	0.214	**0.000**	**<0.0001**	**0.009**	**<0.0001**	0.733	**0.014**	**<0.0001**	0.922	**0.009**	0.153	**<0.0001**	**<0.0001**	**0.001**	0.399	0.864		-0.472
Zn		**0.008**	**0.027**	**0.044**	0.059	0.107	**0.011**	**0.013**	0.475	**0.010**	**0.018**	**0.032**	**0.008**	0.801	0.131	0.053	0.127	0.835	**<0.0001**	0.184	0.485	0.778	**0.001**	**0.003**	0.076	

Values in bold are different from 0 with a significance level α=0.05

Table S2. Correlation matrix (Pearson), *r*-values and *p*-values in *Portulaca oleracea*.

Variables		Salinity	Fresh Weight	Dry Weight	Dry Matter	Fructose	Glucose	Maltose	Oxalic acid	Malic acid	Fumaric acid	Succinic acid	Ascorbic acid	Citric acid	Total Proteins	Violaxan thin	Lutein	b-carotene	P	K	Ca	Mg	Fe	Mn	Na	Zn
													***p*-values**													
Salinity			**-0.955**	**-0.945**	**0.773**	**0.970**	**0.815**	**0.947**	**-0.748**	**-0.913**	**-0.915**	**-0.953**	-0.069	**-0.607**	**-0.845**	-0.105	**-0.594**	0.178	**0.716**	**-0.924**	**-0.882**	-0.190	**-0.955**	-0.417	0.392	**0.857**
Fresh Weight		**<0.0001**		**0.993**	**-0.828**	**-0.960**	**-0.859**	**-0.895**	**0.657**	**0.830**	**0.829**	**0.891**	0.166	0.482	**0.817**	0.178	**0.599**	-0.099	**-0.652**	**0.869**	**0.807**	0.238	**0.906**	0.433	-0.512	**-0.830**
Dry Weight		**<0.0001**	**<0.0001**		**-0.790**	**-0.956**	**-0.875**	**-0.881**	**0.666**	**0.822**	**0.818**	**0.898**	0.234	0.488	**0.816**	0.202	**0.618**	-0.072	**-0.617**	**0.847**	**0.776**	0.239	**0.877**	0.445	**-0.538**	**-0.812**
Dry Matter		**0.001**	**0.000**	**0.000**		**0.847**	**0.829**	**0.705**	-0.383	**-0.607**	**-0.613**	**-0.706**	-0.198	-0.148	-0.492	-0.287	**-0.625**	-0.219	**0.661**	**-0.685**	**-0.586**	-0.193	**-0.791**	-0.388	**0.650**	**0.801**
Fructose		**<0.0001**	**<0.0001**	**<0.0001**	**<0.0001**		**0.917**	**0.901**	**-0.653**	**-0.842**	**-0.842**	**-0.926**	-0.205	-0.459	**-0.743**	-0.247	**-0.690**	-0.038	**0.741**	**-0.855**	**-0.778**	-0.166	**-0.925**	-0.425	**0.565**	**0.905**
Glucose		**0.000**	**<0.0001**	**<0.0001**	**0.000**	**<0.0001**		**0.685**	-0.397	**-0.597**	**-0.596**	**-0.770**	-0.418	-0.189	**-0.582**	-0.333	**-0.668**	-0.277	**0.589**	**-0.631**	**-0.555**	-0.149	**-0.735**	-0.346	**0.793**	**0.830**
Maltose		**<0.0001**	**<0.0001**	**<0.0001**	**0.003**	**<0.0001**	**0.005**		**-0.853**	**-0.946**	**-0.940**	**-0.932**	-0.086	**-0.661**	**-0.760**	-0.184	**-0.678**	0.201	**0.698**	**-0.923**	**-0.808**	-0.187	**-0.920**	**-0.524**	0.257	**0.773**
Oxalic acid		**0.001**	**0.008**	**0.007**	0.159	**0.008**	0.143	**<0.0001**		**0.919**	**0.898**	**0.841**	0.196	**0.864**	**0.652**	0.211	**0.626**	-0.286	**-0.523**	**0.750**	**0.603**	0.069	**0.680**	0.491	0.056	**-0.524**
Malic acid	***r*-values**	**<0.0001**	**0.000**	**0.000**	**0.016**	**<0.0001**	**0.019**	**<0.0001**	**<0.0001**		**0.998**	**0.956**	0.068	**0.806**	**0.749**	0.127	**0.613**	-0.219	**-0.766**	**0.861**	**0.787**	0.020	**0.871**	0.371	-0.106	**-0.789**
Fumaric acid		**<0.0001**	**0.000**	**0.000**	**0.015**	**<0.0001**	**0.019**	**<0.0001**	**<0.0001**	**<0.0001**		**0.954**	0.042	**0.804**	**0.757**	0.098	**0.583**	-0.222	**-0.788**	**0.853**	**0.796**	-0.013	**0.872**	0.324	-0.097	**-0.807**
Succinic acid		**<0.0001**	**<0.0001**	**<0.0001**	**0.003**	**<0.0001**	**0.001**	**<0.0001**	**<0.0001**	**<0.0001**	**<0.0001**		0.224	**0.692**	**0.748**	0.240	**0.702**	-0.049	**-0.747**	**0.847**	**0.756**	0.056	**0.882**	0.406	-0.331	**-0.849**
Ascorbic acid		0.806	0.554	0.401	0.479	0.463	0.121	0.759	0.484	0.809	0.883	0.421		-0.096	-0.063	**0.659**	**0.622**	**0.609**	0.048	-0.082	-0.339	0.004	-0.072	0.335	**-0.556**	-0.115
Citric acid		**0.016**	0.069	0.065	0.598	0.085	0.500	**0.007**	**<0.0001**	**0.000**	**0.000**	**0.004**	0.733		**0.658**	-0.049	0.258	-0.503	-0.496	**0.544**	**0.568**	-0.248	0.488	0.052	0.392	-0.439
Total Proteins		**<0.0001**	**0.000**	**0.000**	0.062	**0.001**	**0.023**	**0.001**	**0.008**	**0.001**	**0.001**	**0.001**	0.822	**0.008**		-0.197	0.190	**-0.546**	-0.403	**0.796**	**0.864**	0.222	**0.744**	0.241	-0.164	**-0.576**
Violaxanthin		0.710	0.527	0.470	0.301	0.374	0.225	0.511	0.450	0.651	0.729	0.389	**0.008**	0.863	0.481		**0.739**	**0.683**	-0.196	-0.011	-0.304	-0.144	0.041	0.347	-0.348	-0.223
Lutein		**0.020**	**0.018**	**0.014**	**0.013**	**0.004**	**0.007**	**0.006**	**0.013**	**0.015**	**0.023**	**0.004**	**0.013**	0.353	0.497	**0.002**		0.466	-0.506	**0.517**	0.212	0.111	**0.557**	**0.647**	**-0.518**	**-0.584**
b-carotene		0.526	0.726	0.798	0.433	0.894	0.318	0.473	0.301	0.434	0.426	0.864	**0.016**	0.056	**0.035**	**0.005**	0.080		-0.169	-0.338	**-0.518**	-0.239	-0.170	0.012	**-0.550**	-0.184
P		**0.003**	**0.008**	**0.014**	**0.007**	**0.002**	**0.021**	**0.004**	**0.046**	**0.001**	**0.000**	**0.001**	0.865	0.060	0.137	0.483	0.054	0.547		**-0.550**	**-0.556**	0.376	**-0.720**	0.054	0.164	**0.933**
K		**<0.0001**	**<0.0001**	**<0.0001**	**0.005**	**<0.0001**	**0.012**	**<0.0001**	**0.001**	**<0.0001**	**<0.0001**	**<0.0001**	0.771	**0.036**	**0.000**	0.968	**0.048**	0.218	**0.033**		**0.924**	0.470	**0.964**	**0.621**	-0.303	**-0.660**
Ca		**<0.0001**	**0.000**	**0.001**	**0.022**	**0.001**	**0.032**	**0.000**	**0.017**	**0.000**	**0.000**	**0.001**	0.217	**0.027**	**<0.0001**	0.271	0.448	**0.048**	**0.032**	**<0.0001**		0.343	**0.911**	0.319	-0.168	**-0.656**
Mg		0.498	0.393	0.391	0.492	0.553	0.597	0.505	0.808	0.944	0.965	0.844	0.988	0.374	0.427	0.609	0.693	0.391	0.168	0.077	0.210		0.323	**0.788**	-0.416	0.193
Fe		**<0.0001**	**<0.0001**	**<0.0001**	**0.000**	**<0.0001**	**0.002**	**<0.0001**	**0.005**	**<0.0001**	**<0.0001**	**<0.0001**	0.799	0.065	**0.001**	0.884	**0.031**	0.546	**0.002**	**<0.0001**	**<0.0001**	0.240		0.494	-0.395	**-0.818**
Mn		0.122	0.107	0.096	0.153	0.114	0.207	**0.045**	0.063	0.173	0.239	0.133	0.223	0.853	0.386	0.205	**0.009**	0.967	0.849	**0.014**	0.246	**0.000**	0.061		-0.437	-0.077
Na		0.148	0.051	**0.039**	**0.009**	**0.028**	**0.000**	0.355	0.843	0.708	0.730	0.229	**0.031**	0.149	0.559	0.204	**0.048**	**0.033**	0.559	0.272	0.551	0.123	0.146	0.103		0.437
Zn		**<0.0001**	**0.000**	**0.000**	**0.000**	**<0.0001**	**0.000**	**0.001**	**0.045**	**0.000**	**0.000**	**<0.0001**	0.682	0.102	**0.025**	0.424	**0.022**	0.512	**<0.0001**	**0.007**	**0.008**	0.491	**0.000**	0.784	0.103	

Values in bold are different from 0 with a significance level α=0.05

Table S3. Correlation matrix (Pearson), *r*-values and *p*-values in *Salsola komarovii*.

Variables		Salinity	Fresh Weight	Dry Weight	Dry Matter	Fructose	Glucose	Raffinose	Oxalic acid	Malic acid	Fumaric acid	Succinic acid	Citric acid	Total Proteins	Violaxanthin	Lutein	b-carotene	P	K	Ca	Mg	Fe	Mn	Na	Zn
		***p*-values**																							
Salinity	*r*-values		-0.272	-0.086	0.494	-0.124	0.410	**-0.703**	0.106	**0.620**	0.485	**-0.949**	**0.947**	-0.003	**0.685**	**-0.920**	**0.801**	**0.990**	**-0.879**	**-0.871**	**-0.670**	-0.359	**-0.785**	**-0.884**	**0.821**
Fresh Weight		0.327		**0.883**	-0.431	-0.241	-0.489	-0.209	**0.529**	-0.348	-0.369	0.280	-0.269	0.406	0.142	0.050	-0.447	-0.190	-0.089	-0.107	-0.340	0.384	-0.207	-0.054	-0.340
Dry Weight		0.759	**<0.0001**		0.032	-0.016	-0.365	-0.162	0.366	0.008	-0.017	0.020	-0.076	0.442	0.181	-0.164	-0.156	-0.029	-0.149	-0.177	-0.318	0.236	-0.278	-0.115	-0.028
Dry Matter		0.061	0.108	0.910		0.479	0.456	0.102	-0.472	**0.807**	**0.786**	**-0.616**	**0.514**	-0.101	0.108	-0.469	**0.719**	0.420	-0.151	-0.154	0.098	-0.342	-0.114	-0.152	**0.721**
Fructose		0.660	0.387	0.955	0.071		**0.536**	0.425	**-0.640**	0.146	0.211	0.041	-0.181	0.258	-0.308	0.255	0.271	-0.211	0.387	0.372	0.433	0.147	0.305	0.427	-0.115
Glucose		0.129	0.064	0.181	0.087	**0.040**		0.111	**-0.664**	0.352	0.272	-0.427	0.320	-0.300	-0.016	-0.174	**0.591**	0.300	-0.090	-0.030	0.166	-0.198	0.029	-0.048	0.269
Raffinose		**0.003**	0.456	0.564	0.718	0.114	0.693		**-0.645**	-0.075	0.071	**0.574**	**-0.625**	-0.428	**-0.792**	**0.703**	-0.367	**-0.782**	**0.891**	**0.928**	**0.938**	0.022	**0.931**	**0.919**	-0.345
Oxalic acid		0.706	**0.043**	0.180	0.076	**0.010**	**0.007**	**0.009**		-0.102	-0.183	0.030	0.155	0.405	**0.589**	-0.283	-0.278	0.217	-0.513	**-0.546**	**-0.738**	-0.005	**-0.612**	-0.505	-0.067
Malic acid		**0.014**	0.204	0.976	**0.000**	0.603	0.198	0.790	0.717		**0.933**	**-0.611**	**0.750**	-0.251	0.438	**-0.650**	**0.603**	**0.553**	-0.419	-0.368	-0.168	**-0.519**	-0.316	-0.349	**0.774**
Fumaric acid		0.067	0.176	0.952	**0.001**	0.451	0.327	0.802	0.514	**<0.0001**		-0.497	**0.642**	-0.205	0.291	**-0.547**	0.492	0.412	-0.272	-0.224	-0.047	-0.351	-0.188	-0.188	**0.686**
Succinic acid		**<0.0001**	0.313	0.944	**0.015**	0.885	0.113	**0.025**	0.915	**0.015**	0.059		**-0.847**	0.045	**-0.540**	**0.910**	**-0.781**	**-0.932**	**0.780**	**0.785**	**0.562**	0.376	**0.707**	**0.799**	**-0.885**
Citric acid		**<0.0001**	0.333	0.789	**0.050**	0.519	0.245	**0.013**	0.582	**0.001**	**0.010**	**<0.0001**		-0.066	**0.717**	**-0.899**	**0.756**	**0.931**	**-0.849**	**-0.815**	**-0.625**	-0.351	**-0.725**	**-0.825**	**0.815**
Total Proteins		0.990	0.133	0.099	0.720	0.353	0.277	0.112	0.134	0.367	0.464	0.873	0.816		0.217	-0.050	0.067	0.067	-0.193	-0.278	-0.437	0.406	-0.422	-0.210	-0.277
Violaxanthin		**0.005**	0.614	0.518	0.703	0.263	0.956	**0.000**	**0.021**	0.103	0.293	**0.038**	**0.003**	0.438		**-0.730**	0.334	**0.727**	**-0.803**	**-0.809**	**-0.788**	-0.011	**-0.827**	**-0.775**	0.495
Lutein		**<0.0001**	0.859	0.559	0.078	0.359	0.536	**0.003**	0.306	**0.009**	**0.035**	**<0.0001**	**<0.0001**	0.860	**0.002**		**-0.602**	**-0.928**	**0.910**	**0.896**	**0.761**	0.343	**0.857**	**0.885**	**-0.823**
b-carotene		**0.000**	0.095	0.578	**0.003**	0.328	**0.020**	0.179	0.315	**0.017**	0.062	**0.001**	**0.001**	0.813	0.224	**0.018**		**0.760**	**-0.521**	**-0.526**	-0.269	-0.321	-0.427	**-0.562**	**0.700**
P		**<0.0001**	0.498	0.918	0.119	0.451	0.277	**0.001**	0.437	**0.032**	0.127	**<0.0001**	**<0.0001**	0.812	**0.002**	**<0.0001**	**0.001**		**-0.916**	**-0.921**	**-0.743**	-0.320	**-0.842**	**-0.935**	**0.796**
K		**<0.0001**	0.752	0.596	0.592	0.155	0.749	**<0.0001**	0.051	0.120	0.327	**0.001**	**<0.0001**	0.491	**0.000**	**<0.0001**	**0.047**	**<0.0001**		**0.985**	**0.929**	0.261	**0.954**	**0.976**	**-0.608**
Ca		**<0.0001**	0.705	0.529	0.585	0.172	0.915	**<0.0001**	**0.035**	0.177	0.422	**0.001**	**0.000**	0.315	**0.000**	**<0.0001**	**0.044**	**<0.0001**	**<0.0001**		**0.943**	0.218	**0.974**	**0.989**	**-0.605**
Mg		**0.006**	0.214	0.248	0.729	0.106	0.554	**<0.0001**	**0.002**	0.551	0.868	**0.029**	**0.013**	0.103	**0.000**	**0.001**	0.333	**0.002**	**<0.0001**	**<0.0001**		0.080	**0.971**	**0.908**	-0.350
Fe		0.189	0.157	0.398	0.212	0.600	0.479	0.938	0.987	**0.047**	0.200	0.167	0.199	0.133	0.970	0.210	0.243	0.245	0.347	0.436	0.776		0.127	0.242	-0.446
Mn		**0.001**	0.459	0.315	0.685	0.269	0.918	**<0.0001**	**0.015**	0.251	0.503	**0.003**	**0.002**	0.117	**0.000**	**<0.0001**	0.113	**<0.0001**	**<0.0001**	**<0.0001**	**<0.0001**	0.651		**0.939**	-0.496
Na		**<0.0001**	0.849	0.684	0.588	0.113	0.864	**<0.0001**	0.055	0.202	0.501	**0.000**	**0.000**	0.453	**0.001**	**<0.0001**	**0.029**	**<0.0001**	**<0.0001**	**<0.0001**	**<0.0001**	0.384	**<0.0001**		**-0.625**
Zn		**0.000**	0.216	0.921	**0.002**	0.684	0.333	0.208	0.812	**0.001**	**0.005**	**<0.0001**	**0.000**	0.318	0.061	**0.000**	**0.004**	**0.000**	**0.016**	**0.017**	0.200	0.095	0.060	**0.013**	

Values in bold are different from 0 with a significance level α=0.05
